# IgG4 Pancreatitis Presenting as a Mass: Unraveling a Diagnostic Illusion

**DOI:** 10.7759/cureus.99052

**Published:** 2025-12-12

**Authors:** Jeffrey Liu, Sindhu Chadalawada, Jagjot Dosanjh, Muhammad Hammami

**Affiliations:** 1 Internal Medicine, University of California San Francisco, Fresno, Fresno, USA; 2 Hepatology, University of California San Francisco, San Francisco, USA; 3 Medicine, University of California San Francisco, San Francisco, USA; 4 Gastroenterology and Hepatology, University of California San Francisco, Fresno, Fresno, USA

**Keywords:** autoimmune pancreatitis, igg4-related pseudotumor, pancreatic biopsy, pancreatic head adenocarcinoma, pancreatitis

## Abstract

Autoimmune pancreatitis is a fibroinflammatory subtype of chronic pancreatitis resulting from aberrant immune responses. Currently, there are two forms of autoimmune pancreatitis: type 1 (T1-AIP) or lymphoplasmacytic sclerosing pancreatitis and type 2, also known as idiopathic duct-centric chronic pancreatitis. T1-AIP is often identified as the pancreatic appearance of immunoglobulin 4 (IgG4)-related disease. T1-AIP commonly appears in the seventh decade of life with a considerable male predominance, frequently associated with elevations in serum IgG4 levels and IgG4-positive cells on tissue biopsy. Type 1 and 2 autoimmune pancreatitis glucocorticoid steroid treatment leads to clinical remission in almost 100% of type 1 and 2 cases. Here, we present a case of an adult who presented with an incidental pancreatic head and tail mass on CT imaging with elevated serum IgG4 levels. He was started on steroid therapy with eventual clinical remission of his disease. This case highlights the rarity of autoimmune pancreatitis and the work-up required to rule out malignancy of the pancreatic mass.

## Introduction

Chronic pancreatitis is a persistent inflammatory disease of the pancreas that leads to tissue damage and subsequent fibrosis [1]. Repeated insult to pancreatic parenchyma can cause leakage and activation of intrapancreatic digestive enzymes. Type 1 autoimmune pancreatitis (T1-AIP) is a distinct subtype of chronic pancreatitis mediated by immunoglobulin 4 (IgG4) [1]. T1-AIP is the pancreatic manifestation of IgG4-related disease [2]. T1-AIP is a rare pancreatitis presentation with an estimated prevalence of 1-2/100,000 people [3,4]. The incidence is underreported, given the similarity in presentation to pancreatic malignancies. Autoimmune pancreatitis is usually diagnosed by tissue sampling post hoc following surgical resection [5]. We present a case of a pancreatic mass, where the biopsy was unclear and required the utilization of other diagnostic criteria to determine the etiology.

## Case presentation

A 67-year-old man with a history of severe aortic stenosis (status post valve replacement), coronary artery disease (status post coronary artery bypass graft), atrial fibrillation, type 2 diabetes mellitus, hypertension, dyslipidemia, and gastroesophageal reflux disease presented with four months of progressive, pressure-like epigastric pain. He also reported early satiety and a 35-pound unintentional weight loss. He denied nausea, vomiting, melena, hematochezia, constipation, or diarrhea. On exam, he was hemodynamically stable without abdominal tenderness or jaundice.

**Figure 1:**
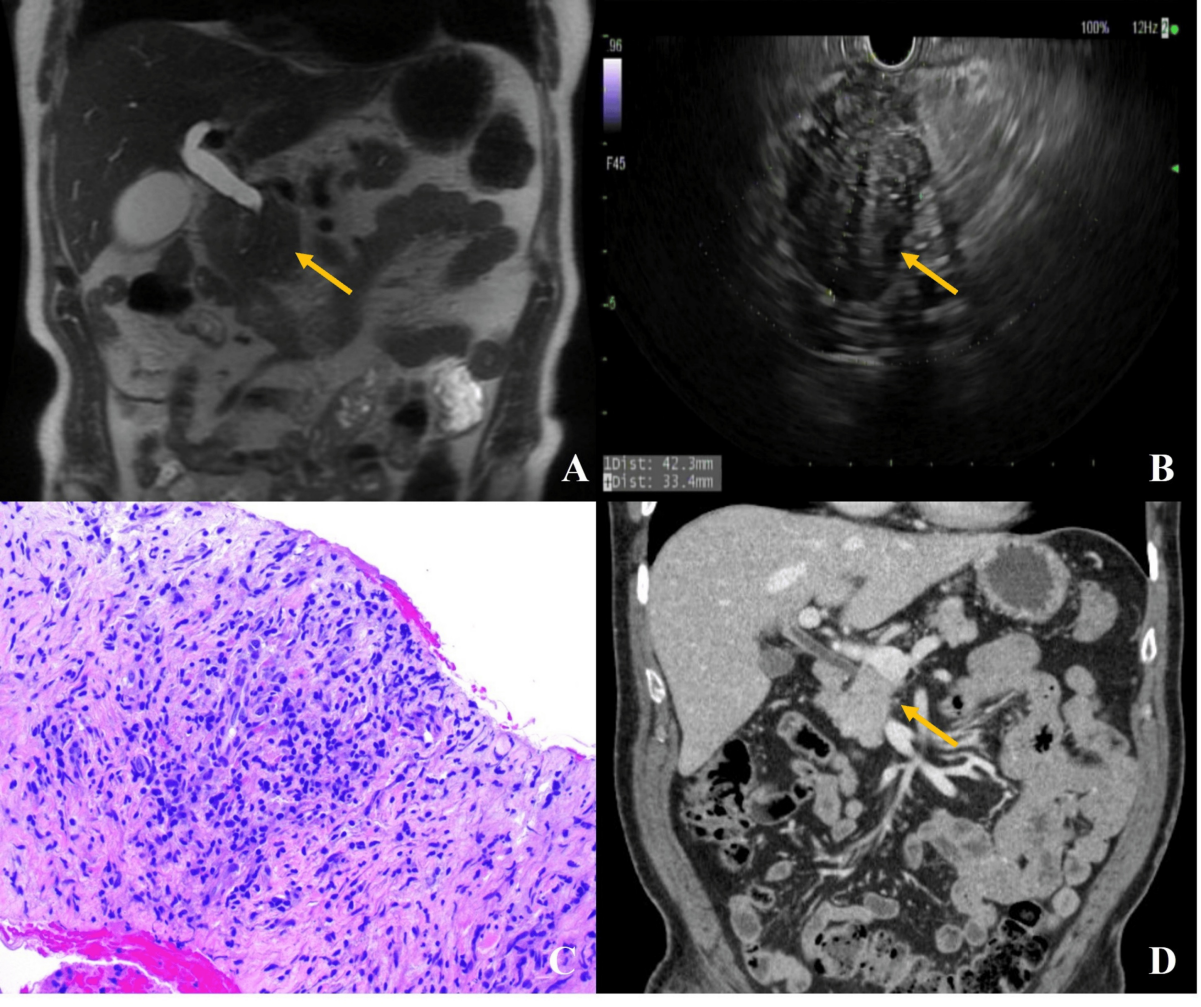
(A) Coronal T2-weighted MRCP image showing mass-like fullness in the pancreatic head with upstream biliary and pancreatic ductal dilatation. (B) Endoscopic ultrasound image showing a hypoechoic, heterogeneous, and irregular mass in the pancreatic head measuring 42.3 × 33.4 mm. (C) Hematoxylin and eosin stain at 100x magnification of pancreatic fine-needle biopsy showing dense lymphoplasmacytic infiltrate with fibrosis, without cytologic atypia or malignant cells. (D) Coronal CT image showing resolution of the pancreatic head mass and normalization of biliary and pancreatic ductal dilatation. Irregular pancreatic head mass highlighted by yellow arrows. MRCP: magnetic resonance cholangiopancreatography

Laboratory evaluation revealed markedly elevated liver-associated enzymes: aspartate aminotransferase (AST), alanine aminotransferase (ALT), alkaline phosphatase (ALP), and total bilirubin (Table 1). Contrast-enhanced CT of the abdomen demonstrated an ill-defined pancreatic head mass measuring at least 3.7 cm, with intrahepatic ductal dilatation and diffuse dilatation of the common bile and pancreatic ducts. Magnetic resonance imaging (MRI) with and without contrast, including magnetic resonance cholangiopancreatography (MRCP), showed a 4.9 × 3.8 × 4.5 cm mass-like fullness in the pancreatic head with homogeneous enhancement similar to the rest of the pancreas, no discrete restricted diffusion, and associated upstream biliary and pancreatic ductal dilatation (Figure 1A).

**Table 1:** Initial serum markers. Units and normal ranges are noted in the right column. “H” denotes abnormal high values.

Test	Patient values	Reference values
Aspartate aminotransferase (AST)	714 (H)	10-40 U/L
Alanine aminotransferase (ALT)	981 (H)	10-50 U/L
Alkaline phosphatase (ALP)	613 (H)	40-129 U/L
Total bilirubin	2.5 (H)	0.1-1.2 mg/dL
Immunoglobulin 4 (IgG4)	300.7 (H)	1-135 mg/dL
Ca antigen 19-9 (CA 19-9)	30.7	0-35 U/mL
Carcinoembryonic antigen (CEA)	1.1	0-5 ng/mL
Alpha fetoprotein (AFP)	1.7	<6.1 ng/mL

Endoscopic ultrasound (EUS) revealed a hypoechoic, heterogeneous, irregular mass in the pancreatic head measuring 42.3 × 33.4 mm (Figure 1B). Fine-needle biopsy (FNB) demonstrated a mixed population of crushed plasma cells, lymphocytes, endothelial cells, and scattered pancreatic acini without evidence of malignancy (Figure 1C). Tumor markers, including alpha fetoprotein (AFP), carcinoembryonic antigen (CEA), and Ca antigen 19-9 (CA 19-9), were within normal limits. Serum IgG4 was significantly elevated (Table 1). The imaging findings and clinical presentation were initially concerning for pancreatic adenocarcinoma. However, the absence of cytologic atypia on biopsy, normal tumor markers, and significantly elevated serum IgG4 raised suspicion for T1-AIP.

Although immunohistochemical staining for IgG4 was not initially performed, the constellation of serologic, radiologic, and histologic findings supported the diagnosis of T1-AIP. The diagnosis was further supported by a favorable response to corticosteroid therapy, which also served as a therapeutic trial. Repeat EUS-FNB after corticosteroid initiation again showed no malignancy, and immunohistochemistry revealed no significant increase in IgG4-positive plasma cells-possibly due to sampling variability or steroid-induced histologic changes.

The patient was started on prednisone 30 mg daily. Within two weeks, his appetite and symptoms improved. The dose was continued for an additional two weeks before initiating a taper of 5 mg per week. Liver chemistries and serum IgG4 normalized (Table 2), and follow-up imaging showed resolution of the pancreatic mass and ductal dilatation (Figure 1D). Repeat EUS-FNB confirmed benign findings.

**Table 2:** Serum markers after 4 weeks of prednisone. Units and normal ranges are noted in the right column.

Test	Patient values	Reference values
Aspartate aminotransferase (AST)	21	10-40 U/L
Alanine aminotransferase (ALT)	24	10-50 U/L
Alkaline phosphatase (ALP)	50	40-129 U/L
Total bilirubin	0.9	0.1-1.2 mg/dL
Immunoglobulin 4 (IgG4)	99.5	1-135 mg/dL

At follow-up, the patient remained asymptomatic. Liver tests remained normal (Table 3), and no relapse occurred. He continues to be monitored for recurrence or signs of systemic IgG4-related disease. No immunosuppressive maintenance therapy has been necessary to date.

**Table 3:** Serum markers at the 6-month follow-up. Units and normal ranges are noted in the right column.

Test	Patient values	Reference values
Aspartate aminotransferase (AST)	18	10-40 U/L
Alanine aminotransferase (ALT)	23	10-50 U/L
Alkaline phosphatase (ALP)	56	40-129 U/L
Total bilirubin	0.7	0.1-1.2 mg/dL

## Discussion

Chronic pancreatitis can be classified through the TIGAR-O system: Toxic-Metabolic, Idiopathic, Genetic, Autoimmune, Recurrent-Severe acute pancreatitis, and Obstructive etiologies [6]. The most common etiologies of chronic pancreatitis include alcohol-induced, tobacco-related, hypertriglyceridemia-associated, cystic fibrosis, and vascular disease. These etiologies can be differentiated from AIP through age of presentation, detailed history-taking, and early diagnostic imaging.

AIP is a rare, steroid-responsive, chronic fibroinflammatory condition that can mimic pancreatic cancer. It accounts for fewer than 5%-10% of all pancreatitis cases in the United States and is estimated to affect 1-2 per 100,000 individuals [3,7]. It is usually diagnosed at the seventh decade of life with a predominant male-to-female distribution of 3:1 [7,8]. AIP is subclassified into type 1 and type 2 based on histopathology and systemic involvement [1,2]. T1-AIP, the IgG4-related variant, is part of a broader systemic IgG4-related disease spectrum, which may involve the biliary tract, salivary glands, kidneys, lungs, and lymph nodes [1,2,9]. T1-AIP typically affects older men and often presents with painless jaundice, abdominal discomfort, weight loss, or a pancreatic mass [1,2]. It is histologically characterized by lymphoplasmacytic infiltration, storiform fibrosis, and obliterative phlebitis, with dense IgG4-positive plasma cell infiltration [4]. Diagnostic criteria include (1) imaging findings of diffuse or focal pancreatic enlargement or ductal strictures, (2) serum IgG4 elevation (>135 mg/dL), (3) histologic confirmation with IgG4+ plasma cells or characteristic architecture, (4) evidence of extra-pancreatic IgG4-related disease, and (5) response to steroids [1,2]. Distinguishing AIP from pancreatic adenocarcinoma is critical but often challenging. A dramatic response to corticosteroids is a hallmark of AIP and can serve both diagnostic and therapeutic purposes.

Poor response to steroids would be highly suspicious for other etiologies, such as pancreatic malignancy [8]. However, relapse does not necessarily raise suspicion for pancreatic malignancy since repeat biopsy studies would likely find a reversal of initial histological findings [10]. In these patients who relapse on steroid therapy, the current consensus is to manage them with immunomodulators such as azathioprine and 6-mercaptopurine [11].

## Conclusions

T1-AIP emphasizes the importance of a multi-disciplinary approach in coordinating management. Early consultation and coordination with General Surgery, Oncology, and Advanced Endoscopy can improve patient outcomes and morbidity if T1-AIP is excluded from the differential diagnosis. Utilizing the HISORt criteria can provide diagnostic guidance when delineating between T1-AIP and pancreatic malignancies. Continued monitoring and close follow-up remain a cornerstone in the long-term management of patients with T1-AIP, given the variability in presentation in patients with IgG4-related disease.
